# Monitoring indices of bone inflammatory activity of the jaw using SPECT bone scintigraphy: a study of ARONJ patients

**DOI:** 10.1038/s41598-020-68428-x

**Published:** 2020-07-09

**Authors:** Hironobu Hata, Tomoka Kitao, Jun Sato, Takuya Asaka, Noritaka Ohga, Kenji Imamachi, Kenji Hirata, Tohru Shiga, Yutaka Yamazaki, Yoshimasa Kitagawa

**Affiliations:** 1 Department of Dentistry and Oral Surgery, Hokkaido Cancer Center, 3-54, Kikusui4-Jyo 2-Tyoume, Shiroishi-Ku, Sapporo, Japan; 2 Department of Radiology, Hokkaido Cancer Center, 3-54, Kikusui4-Jyo 2-Tyoume, Shiroishi-Ku, Sapporo, Japan; 3 Oral Diagnosis and Medicine, Department of Oral Pathobiological Science, Faculty of Dental Medicine, Hokkaido University, Nishi 7-Tyoume Kita13-Jyo, Kita-Ku, Sapporo, Japan; 4 Gerodontology, Department of Oral Health Science, Faculty of Dental Medicine, Hokkaido University, Nishi 7-Tyoume Kita13-Jyo, Kita-Ku, Sapporo, Japan; 5 Department of Diagnostic Imaging, Graduate School of Medicine, Hokkaido University, Nishi 7-Tyoume Kita15-Jyo, Kita-Ku, Sapporo, Japan

**Keywords:** Oral diseases, Chronic inflammation

## Abstract

Development of quantitative analysis software has enabled application of several standardised uptake values (SUV) for bone analysis in single photon emission computed tomography (SPECT). The present retrospective study aimed to develop a reliable method of monitoring bone inflammatory activity in antiresorptive agent-related osteonecrosis of the jaw (ARONJ) using SPECT quantitative analysis software. Fifteen ARONJ patients underwent SPECT before and after anti-inflammatory therapy. We calculated the mean maximum SUV (SUVmax) of the bilateral cranial bones using quantitative analysis software and used this as the control [C]. We attempted to adjust the SUVmax of the lesion [L] as follows: *adjusted SUVmax* (*aSUVmax*) = [*L*] − [*C*]*.* The optimum threshold to calculate the metabolic bone volume (MBV) (cm^3^) was [C] + 3. The threshold values obtained for each case were input to calculate MBV at each osteomyelitis site. Retrospectively, we compared aSUVmax and MBV of each patient’s ARONJ before and after anti-inflammatory therapy. The patients’ high aSUVmax or large MBV of the ARONJ reduced rapidly, reflecting individual clinical findings after treatment. Application of SPECT quantitative analysis software to monitor bone inflammatory activity in ARONJ could improve the prognosis-deciding abilities of clinicians and enable them to treat ARONJ effectively.

## Introduction

With the presence of ageing populations and longer life spans, osteoporosis is increasingly becoming a global epidemic. Currently, it has been estimated that more than 200 million people suffer from osteoporosis worldwide^1^. Metastatic bone disease affects between 280,000 and 330,000 people in the United States^2^. Consequently, the number of patients using bone-targeting agents has increased, and the incidence of antiresorptive agent-related osteonecrosis of the jaw (ARONJ) is also increasing. ARONJ is intractable and rarely cured spontaneously when it occurs. ARONJ significantly affects the quality of life (QoL), a detriment that increases with worsening osteonecrosis^3^. Although imaging modalities such as panoramic radiograph, computed tomography (CT), and nuclear medicine provide important diagnostic information, the role of diagnostic imaging for ARONJ has not been completely established^4^. The standard treatment of ARONJ is still controversial, particularly when comparing non-surgical and surgical approaches. The incidence of ARONJ in the osteoporosis patient population is very low and is estimated at 1–90 per 100,000 patient-years of exposure. In the oncology patient population, the incidence of ARONJ appears to be related to the dose and duration of exposure, and the prevalence has been estimated to be as high as 18.6%^5^.

Although the developmental mechanism of ARONJ has not been completely established, it is known that antiresorptive agents alone do not cause bone necrosis, but when combined with trauma such as tooth extraction or inflammation/infection from periodontal or periapical disease, bone necrosis can occur. Bone exposure propagates infection and inflammation in a positive feedback loop to increase disease severity or extent^6^. The main risk factor and driver for the development of ARONJ is the presence or occurrence of local infections within the jawbone^7^. ARONJ does not simply refer to the histopathological necrotic bone; rather, it represents the clinical condition of the change in bone inflammation with or without sequestrum. The control of local infection and inflammation leads to suppression and management of ARONJ. Unfortunately, no objective index that can assess the anti-inflammatory effect in ARONJ has been described to date.

Bone scintigraphy has very high sensitivity for the detection of ARONJ and provides visualisation of the whole skeleton. Some studies have reported the usefulness of bone scintigraphy and single photon emission computed tomography (SPECT) and SPECT/CT in diagnostic bisphosphonate-related osteonecrosis of the jaw^8,9^. A standardised uptake value (SUV) has been previously applied to the bone SPECT to treat prostate cancer patients with bone metastases^10^. However, no SPECT quantitative analysis software has been applied to accurately monitor bone inflammatory activity in ARONJ by comparing intra-patient data at any two time points. Therefore, the purpose of this case series was to evaluate the usefulness of SUV evaluation in bone SPECT to assess the activity of ARONJ.

## Results

### Pilot study

**Figure 1:**
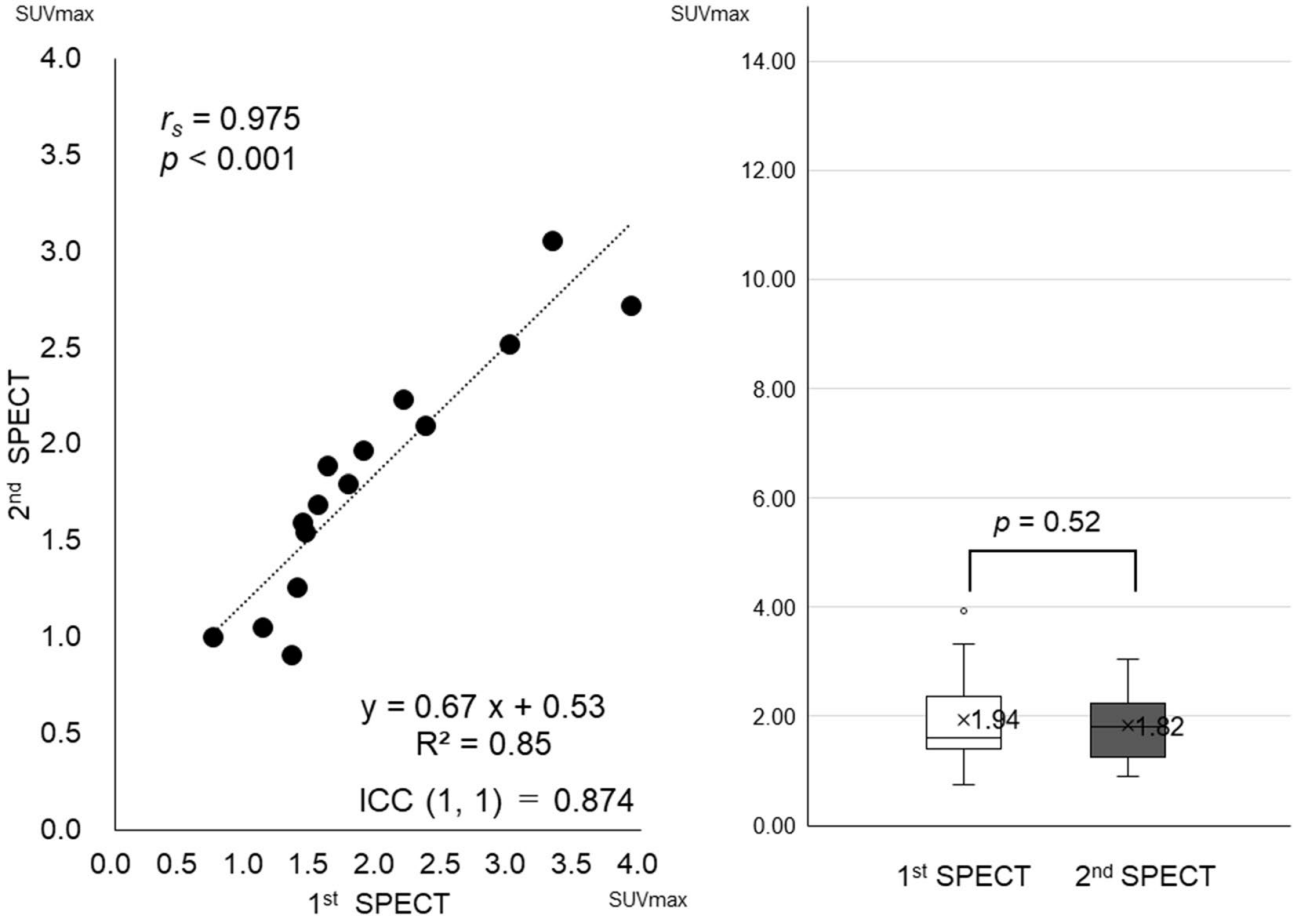
Plotted graphics and the box-and-whisker plot of SUVmax at the 1st and 2nd SPECT analysis of cranial bone in each patient; high reproducibility in cranial bone and high accuracy of software was confirmed {ICC (1,1) = 0.87 and R^2^ = 0.85. It was clear that SUVmax of the cranial bone was not affected by anti-inflammatory therapy (*p* = 0.52).

Following the pilot SPECT analysis of maximum standardised uptake value (SUVmax) of the cranial bone, the right and left SUVmax was almost of the same value at the 1st and 2nd intra-patient SPECT imaging, and these showed a significant correlation (*r*_*s*_ = 0.975, *p* < 0.001). Moreover, it became clear that SUVmax of the cranial bone had no laterality without the influence of odontogenic infection (data not shown). It was also clear that the high reproducibility of SUVmax of the cranial bone at the 1st and 2nd intra-patient SPECT imaging {ICC (1, 1) = 0.87 and R^2^ = 0.85}, high accuracy of the software, and SUVmax of cranial bone were not affected by anti-inflammatory therapy including HBO (*p* = 0.52) (Fig. 1). In addition, the SUVmax of the cranial bone at the 1st and 2nd intra-patient SPECT imaging showed considerable scattering among patients. 1st/2nd Mean ± standard deviation [minimum, maximum] = 1.94 ± 0.88 (0.75, 3.92)/1.82 ± 0.64 (0.91, 3.06) (Table 1).

**Table 1:** Adjusted SUVmax of patients in SPECT imaging analysis.

No.	First SPECT imaging			Second SPECT imaging		
	Osteomyelitis SUVmax [L]	Cranial bone SUVmax [C]	Adjusted SUVmax = [L] − [C]	Osteomyelitis SUVmax [L]	Cranial bone SUVmax [C]	Adjusted SUVmax = [L] − [C]
1	14.79	1.77	13.02	14.54	1.80	12.74
2	20.82	1.45	19.37	14.37	1.55	12.82
3	10.98	0.75	10.23	13.56	1.00	12.56
4	17.33	2.20	15.14	14.05	2.23	11.82
5	12.33	1.61	10.71	8.51	1.89	6.62
6	16.59	1.55	15.05	14.63	1.69	12.95
7	9.01	1.39	7.62	10.32	1.26	9.06
8	18.20	2.37	15.83	7.90	2.10	5.80
9	13.06	1.43	11.64	12.36	1.60	10.76
10	10.51	1.12	9.39	13.06	1.05	12.01
11	26.06	1.34	24.72	14.53	0.91	13.62
12	6.15	3.00	3.15	5.19	2.52	2.67
13	11.68	3.33	8.35	8.38	3.06	5.32
14	4.97	1.89	3.08	5.02	1.97	3.05
15	9.64	3.92	5.71	8.17	2.72	5.45
Mean	13.47	1.94	11.53	10.97	1.82	9.15

*SPECT* single photon emission computed tomography, *SUV* standardised uptake value, *L* lesion part, *C* control part.

We decided to use the SUVmax of the cranial bone as a control and perform an inter-patient comparison of the SUVmax of the ARONJ after adjusting for individual differences in the SUVmax of healthy bone.

### Adjusted SUVmax and metabolic bone volume of the patients

**Figure 2:**
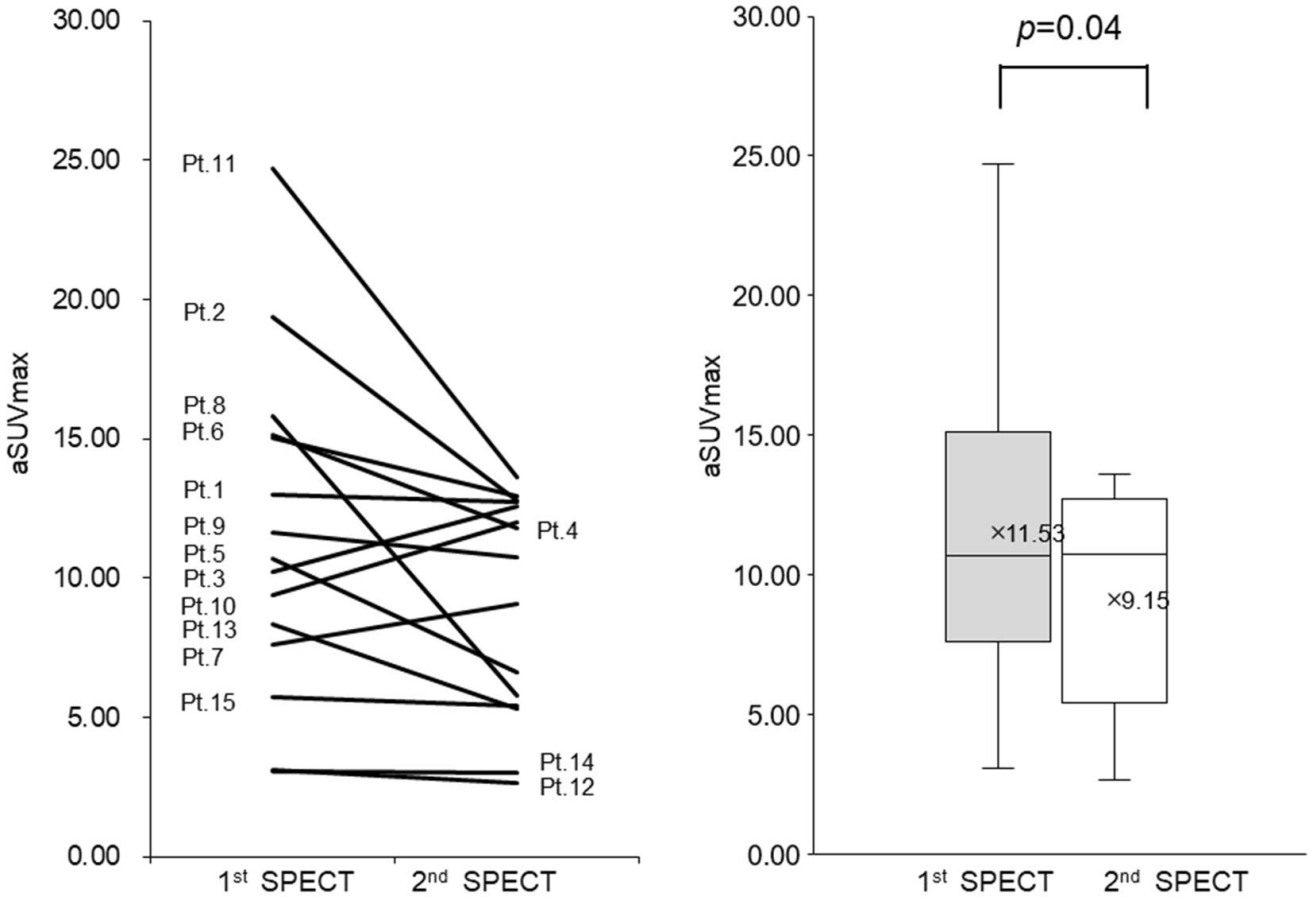
Transition line graph and box-and-whisker plot of aSUVmax at the 1st and 2nd SPECT imaging of each patient; patients who had a high aSUVmax of 15 or more in the 1st round showed a marked decrease in the 2nd round (patients: 2, 4, 6, 8, and 11), but patients who had a low aSUVmax of less than 6 in the 1st round showed little change in the 2nd round (patients: 12, 14, and 15). The overall aSUVmax was significantly reduced after anti-inflammatory therapy (*p* = 0.04).

**Figure 3:**
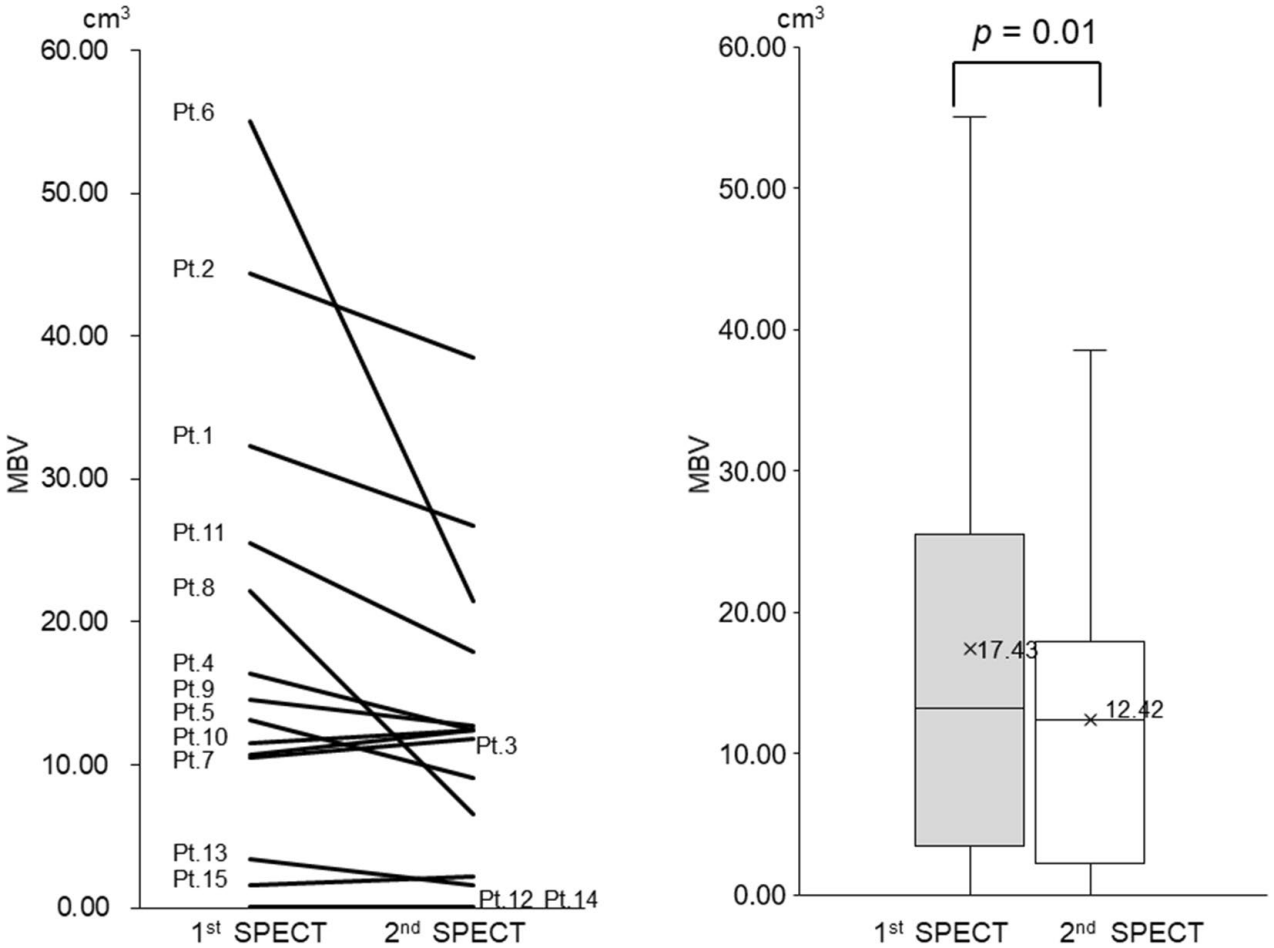
Transition line graph and box-and-whisker plot of MBV at the 1st and 2nd SPECT imaging of each patient; patients who had a high MBV of 20 cm^3^ or more in the 1st round showed a marked decrease in the 2nd round (patients: 1, 2, 6, 8, and 11), but patients who had a low MBV of less than 2 cm^3^ in the 1st round, showed little change in the 2nd round (patients: 12, 14, and 15). Regarding patients 12 and 14, they showed almost less than the limit of detection because even at pre-and post-treatment, the overall MBV was significantly reduced after anti-inflammatory therapy (*p* = 0.01) and the mean MBV of the 1st/2nd round of SPECT was 17.43 ± 16.16 cm^3^/12.42 ± 10.59 cm^3^.

We calculated each patient’s aSUVmax and metabolic bone volume (MBV) before and after the anti-inflammatory treatment using GI-BONE and the previously mentioned equation (Tables 1 and 2). Additionally, the transition line graph and box-and-whisker plot of aSUVmax and MBV before and after the anti-inflammatory treatment of each patient are shown (Figs. 2 and 3). The overall aSUVmax was significantly reduced after the anti-inflammatory treatment. The mean aSUVmax of the 1st/2nd time of SPECT = 11.5 ± 5.9/9.2 ± 3.9 (*p* = 0.04) (Fig. 2).

**Table 2:** Threshold and metabolic bone volume (MBV) in SPECT imaging analysis.

No.	First time SPECT		Second time SPECT	
	Threshold = [C] + 3 (SUV)	MBV (cm^3^)	Threshold = [C] + 3 (SUV)	MBV (cm^3^)
1	4.77	32.36	4.80	26.74
2	4.45	44.39	4.55	38.48
3	3.75	10.52	4.00	11.88
4	5.20	16.36	5.23	12.46
5	4.61	13.17	4.89	9.09
6	4.55	55.05	4.69	21.48
7	4.39	10.70	4.26	12.49
8	5.37	22.17	5.10	6.56
9	4.43	14.57	4.60	12.78
10	4.12	11.53	4.05	12.42
11	4.34	25.52	3.91	17.97
12	6.00	0.04	5.52	0.04
13	6.33	3.44	6.06	1.61
14	4.89	0.07	4.97	0.07
15	6.92	1.63	5.72	2.24
Mean	4.94	17.43	4.82	12.42

*SPECT* single photon emission computed tomography, *SUV* standardised uptake value, *C* control part, *MBV* metabolic bone volume.

Similarly, overall MBV was significantly reduced after the anti-inflammatory treatment. The mean MBV of the 1st and 2nd time of SPECT = 17.4 ± 16.2 cm^3^/12.4 ± 10.6 cm^3^ (*p* = 0.01) (Fig. 3).

### Comparison of aSUVmax and MBV with clinical course

We compared the clinical course of OMJ with the change in aSUVmax and MBV between the 1st and 2nd time of SPECT for each patient. Considering that the highest SUVmax of the cranial bone as a control value was about 4, the patients who had a low aSUVmax of less than 6 at 1st time (patients: 12, 14, and 15) were regarded to display an extremely low inflammatory activity for ARONJ at baseline (Fig. 2). Similarly, the patients who had low MBV of less than 2.0 cm^3^ at the 1st time of SPECT (patients: 12, 14, and 15) were considered to have almost no volume showing inflammatory activity at baseline (Fig. 3). These 3 patients had been using antibiotics for a prolonged period of several months before baseline, and while bone exposure was observed, infectious findings such as drainage were not observed (Stage 1). However, patients who had a refractory infection at the baseline and had a high aSUVmax of 15.0 or more (patients: 2, 4, 6, 8, and 11) and a large MBV of 20.0 cm^3^ or more (patients: 1, 2, 6, 8, and 11) at baseline showed a sharp decrease in both values after anti-inflammatory treatment. On the other hand, the two cases (patient 3 and patient 10) whose aSUVmax difference exceeded 2 (2.34/2.62), showed a worsened C-reactive protein (CRP) level (pre/post), with patient 3 having 0.41 µg/mL/1.26 µg/mL and patient 10 having 1.07 µg/mL/4.18 µg/mL, respectively. In particular, Patient 10, who did not use an antibiotic during anti-inflammatory therapy, showed an increase in the amount of drainage from the sequestrum area at the 2nd time of SPECT, following which the infection became worse. The MBV also increased in these two patients (patient 3 and patient 10), but only slightly (1.36 cm^3^/0.89 cm^3^).

**Figure 4:**
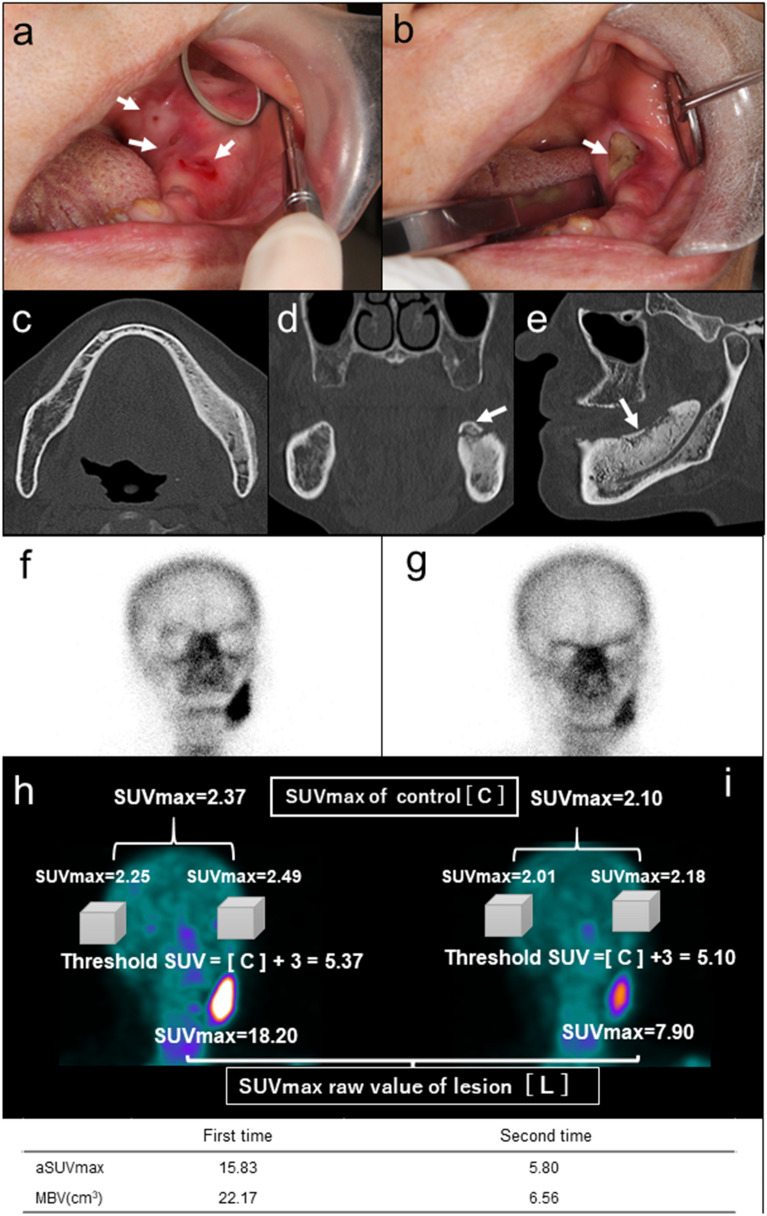
A representative case (patient 8), where there was intense swelling with redness in the left lower gingiva and discharge of pus from several fistulas at the baseline (*arrows*) (**a**). After anti-inflammatory therapy, the gingival swelling improved. The unification of fistula and exposure of the sequestrum was observed but pus was not observed (*arrow*) (**b**). CT at the baseline showed marked osteosclerotic changes of the lower left mandibular body and bone apposition on the outer surface of the cortical bone (**c**). Sequestrum was found in the coronal and oblique sagittal reconstruction images (*arrow*) (**d**, **e**). After pre-operative treatment, the sequestrum was removed by conservative surgical treatment and the patient reported no further recurrence. The 1st planar image showed a strong uptake in the lower left mandible (**f**) that remarkably decreased in the 2nd image (**g**). This 2nd image could not quantitatively represent the uptake intensity and volume. SPECT reconstructed the image by GI-BONE. The threshold for the volume of interest (VOI) was determined to be the value of the mean SUVmax of the right and left cranial bones plus three (1st/2nd round of SPECT) = (5.37/5.10). In the 1st SPECT imaging (**h**), adjusted SUVmax (aSUVmax) was caluculated SUVmax of lesion [L =18.20] - SUVmax of control [C =2.37] = 15.83. In the 2nd SPECT imaging (**i**), despite the short duration of 4.9 weeks, aSUVmax decreased sharply from 15.83 to 5.80. MBV also decreased sharply from 22.17 to 6.56 cm^3^.

As a representative case, we presented the clinical course of patient 8 with CT images, planar image of bone scintigraphy, and SPECT reconstruction images by GI-BONE (Fig. 4).

## Discussion

In this study, we proposed a method to calculate a data standardisation equation using SPECT bone scintigraphy fixed-quantity analysis software, which involves comparisons among patients in uptake intensity and volume. Considering the individual differences in normal bone SUVmax, the direct interpatient comparisons of raw SUVmax data of the lesions were inappropriate.

Previously, to assess the severity of OMJ, Fukumitsu et al*.* tried to quantify the uptake value of planar bone scintigraphy by proposing another equation. In their semiquantitative analysis, a region of interest (ROI) was manually placed over the area with the high accumulation of ^99m^Tc-HMDP in the lower jaw. A symmetrical ROI was then placed over the contralateral normal region of the lower jaw as the control^8^. They calculated the ‘uptake ratio’ by dividing the count of the lesion by the count of the control. When we applied this method to our study, (divided the SUVmax of the lesion area by the SUVmax in the control area) it eliminated the inherent significance of the SUV already standardised by the dose of radioactivity (Bq) and body weight (g). Moreover, the contralateral jaw used in the previous study as a control area often showed alveolitis caused by other odontogenic infections. In such a case, the uptake ratio of the lesion may be calculated as a lower value. Therefore, we proposed an equation to standardise the SUVmax of each patient’s lesion part using the subtraction method.

According to the pilot study results, neither was the cranial bone affected by odontogenic inflammation or HBO, nor did it show laterality among the patients. These results supported our idea that cranial bone was suitable as a control in our SPECT imaging range, therefore, we used SUVmax of bilateral cranial bones as the control. Similarly, considering the individual differences in normal bone SUVmax, we found that absolute thresholds are undesirable and applied relative thresholds for the calculation of MBV. Although the number of cases is the same, compared with the Fukumitsu’s semiquantitative analysis method of planar images^11^, our method of using the quantitative analysis SPECT software (seen as CT images) has improved the amount of image information to be processed, enhanced the reproducibility, and facilitated the evaluation of the upper jaw. In addition to quantifying the intensity of bone inflammation as SUV, our method is excellent in quantifying the tracer accumulation volume above a certain threshold SUV as MBV.

The mean aSUVmax and mean MBV of all cases decreased significantly after anti-inflammatory therapy, despite the short period (within 6 weeks) of imaging. These results strongly suggested that aSUVmax and MBV could be accurate indicators for monitoring the anti-inflammatory effect in ARONJ. Discussion of the following individual cases supported this opinion.

According to the results of comparison of aSUVmax and MBV with clinical course, 3 cases (patients 12, 14, and 15) may have shown a low bone inflammatory activity at the baseline. As a result, it is possible that pre-operative anti-inflammatory treatment was not required. However, patients who had a high aSUVmax of 15 or more or a large MBV of 20 cm^3^ or more at baseline (patients: 1, 2, 4, 6, 8, and 11) benefited from anti-inflammatory treatment. SPECT quantification data reflected individual clinical findings. With the exception of two patients (patient 2 and patient 10), none of the CRP values presented in this study correlated with the clinical data. Therefore, we observed that clinical findings and blood tests for osteomyelitis may not always match. There was no difference in the white blood cell (WBC) count with or without osteomyelitis; even for acute osteomyelitis, WBC count has often been shown to be normal^12,13^. The erythrocyte sedimentation rate (ESR) and CRP are often elevated in osteomyelitis, but lack specificity in the absence of other imaging and microbiological data^14^. These facts make it difficult to establish a gold standard for assessing osteomyelitis activity.

Regarding OMJ, the region with the highest uptake in bone SPECT very likely represents the area where the highest inflammatory activity can be expected^15^.

Furthermore, Modabber et al*.* has shown that there is a significant correlation between the pathological range of osteomyelitis of the mandible and the range of SPECT/CT accumulation^16^. In this study, SUVmax in SPECT showed the highest inflammatory activity, and monitoring SUVmax was equal to monitoring the inflammatory activity of OMJ. Moreover, by using the appropriate thresholds SUV, MBV was calculated and was expected to represent the volume of osteomyelitis in ARONJ.

There are several limitations to this study. Firstly, it was an exploratory retrospective study, which was performed in a single facility with a small number of cases and changes in aSUVmax. In addition, since comparisons with the untreated or reference group were not performed, we could not deny the possibility that these changes in aSUVmax and MBV may simply reflect the natural history of ARONJ. Secondly, we could not compare our measurement results because the gold standard for assessing bone inflammatory activity has not been established to date. Moreover, we did not establish a uniform anti-inflammatory period until the baseline and did not standardise the use and type of antibiotics.

In general, SPECT quantitative analysis software is ideally used in SPECT/CT device for accurate attenuation correction of the nuclear medicine image data, but this device was not available. We could not investigate whether SUVmax or SUVpeak was superior as a quantitative imaging biomarker. In the future, we need to conduct multi-centre studies to enable harmonisation of the devices and to standardise the staging of the severity of OMJ.

This is the first study that applied SPECT quantitative analysis software to evaluate the uptake intensity and volume of bone inflammatory activity in ARONJ at two time points of SPECT imaging, before and after anti-inflammatory treatment. We proposed two equations for the adjusted SUVmax and threshold SUV to determine MBV. The patient's high aSUVmax or large MBV reduced rapidly, reflecting individual clinical findings after treatment. Multi-centre studies are required to confirm whether this method is useful in other devices and at other facilities. This could improve the prognosis-deciding abilities of clinicians and enable them to approach the treatment of ARONJ more effectively, while increasing the viability of SPECT imaging.

## Material and methods

### Patients and staging of ARONJ

This is an exploratory study; therefore, sample size was not calculated. Fifteen ARONJ patients who presented at Hokkaido University Hospital’s Oral Diagnosis and Medicine Department from 1 July 2008 to 31 September 2014 were enrolled in this retrospective clinical study. The inclusion criteria were: (1) ARONJ patients who completed the following anti-inflammatory treatment and (2) were able to undergo SPECT imaging twice; once before and once after the treatment. The exclusion criteria were: ARONJ Stage 0 patients with no apparent necrotic bone exposure and no medical history of head and neck radiation therapy. The patients included 2 men and 13 women with a median age of 74.9 years (range: 58–90 years). ARONJ was staged as follows; Stage 1 (n = 3), Stage 2 (n = 7), and Stage 3 (n = 5). Stage 0 ARONJ patients were excluded. The patients’ diseases were as follows: cancer (n = 4), rheumatoid arthritis (n = 6), osteoporosis (n = 4), and nephrotic syndrome (n = 1) Five patients had diabetes mellitus, five patients were receiving systemic steroid therapy, and three patients were receiving methotrexate. No patients had anaemia or Paget's disease of the bone; 8 out of 15 patients who used anti-resorptive drug for more than 48 months had the risk of developing ARONJ (Table 3).

**Table 3:** Characteristics of all patients.

No.	Sex	Age	ARONJ stage	Jaw	Primary disease	ARD	Administration route of ARD	Medication period of ARD (months)	Risk factor for ARONJ
1	F	90	2	Mandible	OP	Risedronate	Intraoral	6	DM
2	F	79	3	Mandible	OP	Risedronate	Intraoral	24	DM
3	F	81	2	Mandible	OP	Risedronate	Intraoral	36	none
4	F	79	2	Mandible	OP	Alendronate	Intraoral	20	DM
5	M	75	3	Mandible	BM (PC)	Zoledronate	Intravenous	22	DM
6	F	67	3	Maxilla	BM (BC)	Zoledronate	Intravenous	66	none
7	F	58	2	Mandible	BM (BC)	Zoledronate	Intravenous	53	none
8	F	76	2	Mandible	BM (BC)	Zoledronate	Intravenous	46	none
9	F	78	3	Maxilla	NPS	Alendronate	Intraoral	66	Steroid
10	M	80	2	Mandible	RA	Alendronate	Intraoral	48	Steroid
11	F	78	3	Mandible	RA	Risedronate	Intraoral	36	Steroid
12	F	63	1	Mandible	RA	Alendronate	Intraoral	60	MTX
13	F	76	2	Mandible	RA	Risedronate	Intraoral	60	Steroid
14	F	77	1	Mandible	RA	Alendronate	Intraoral	120	MTX
15	F	66	1	Maxilla	RA	Risedronate	Intraoral	120	Steroid MTX

*M* male, *F* female, *ARONJ* antiresorptive agent-related osteonecrosis of the jaw, *ARD* anti-resorptive drug, *BC breast cancer, BM* bone metastasis, *DM* diabetes mellitus, *MTX* methotrexate, *NPS* nephrotic syndrome, *OP* osteoporosis, *PC* prostate cancer, *RA* rheumatoid arthritis.

The anti-resorptive agent-related osteonecrosis of the jaw together with the bisphosphonates and anti-RANKL antibody (denosumab) related osteonecrosis of the jaw has been called ARONJ^17^. The American Association of Oral and Maxillofacial Surgeons (AAOMS) Position Paper advocates the designation of medication-related osteonecrosis of the jaw (MRONJ), including jaw bone necrosis caused by angiogenesis inhibitors^18^. Our patients were diagnosed with stage MRONJ/ARONJ according to the diagnostic criteria in the 2014 AAOMS Position Paper as follows; stage 0: no clinical evidence of necrotic bone with non-specific clinical findings, radiographic changes, and symptoms; stage 1: exposed and necrotic bone or fistulae that project into the bone in asymptomatic patients with no evidence of infection; stage 2: exposed and necrotic bone or fistulae that project into the bone, associated with infection evidenced by pain and erythema in the region of the exposed bone with or without purulent drainage; stage 3: exposed and necrotic bone or fistulae that project into the bone in patients with pain, infection, and one or more of the following; exposed and necrotic bone extending beyond the region of alveolar bone, i.e. , the inferior border and ramus of the mandible and the maxillary sinus and zygoma in the maxilla; pathologic fracture; extra-oral fistula; oral antral/nasal communication; or osteolysis extending to the inferior border of the mandible or the sinus floor.

### Anti-inflammatory treatment and timing of SPECT imaging

Antibiotic therapy and topical irrigation were used as needed, and 20 HBO sessions were provided for all cases. No anti-inflammatory or bone resorption inhibitors were used in any patient; however, anti-diabetic drugs were used in 5 patients who had a history of diabetes during anti-inflammatory treatment.

Before the start of anti-inflammatory therapy, the interval of SPECT imaging was set at approximately 6 weeks and an average interval between two image scans of 5.8 ± 2.4 weeks.

### Bone SPECT and quantitative analysis

Bone SPECT was performed 4 h after an intravenous injection of 555 MBq technetium-99 m hydroxy methylene diphosphonate (^99m^Tc-HMDP, Nihon Medi-Physics Co., Ltd., Tokyo, Japan) using a SPECT system (ECAM, Siemens Healthcare, Erlangen, Germany). The SPECT images were acquired as follows; low-energy high-resolution collimator, step-and-shoot mode with 30 s per step and 72 steps per detector, 360 degrees, a matrix size of 128 × 128, a pixel size of 3.30 mm, and energy window at 140 keV ± 10%. The 1st SPECT was performed within 1 week before the start of treatment and the 2nd SPECT was performed within 1 week after the end of the treatment.

The SPECT images were reconstructed using the ordered-subsets expectation maximisation (OSEM) method with two iterations and 12 subsets. Images were smoothed with a 3D spatial Gaussian filter at 8.4 mm at full width and half maximum (FWHM). Becquerel Calibration Factor (BCF), a numeric factor used to convert a pixel value into the SUV, was measured using a cylindrical phantom filled with uniform ^99m^Tc solution. BCF was determined at 12,953 Bq/(counts/seconds). The SUV was calculated as^19^;

$$[{\text{BCF}}\,\left( {{\text{Bq/cps}}} \right) \times {\text{Body}}\,{\text{weight}}\,\left( {\text{g}} \right) \times {\text{SPECT}}\,{\text{count}}\,{\text{density}}\,({\text{count/cc}})]/[{\text{Scan}}\,{\text{duration}}\,\left( {\sec } \right) \times {\text{Injected}}\,{\text{activity}}\,({\text{Bq}}) $$

GI-BONE (AZE Co., Ltd., Tokyo, Japan), a vendor-neutral software for bone SPECT was used to calculate the BCF, SUVmax, and MBV (cm^3^).

The software computed the SUVmax for the bilateral cranial bones and the ARONJ lesion by setting the volume of interest (VOI). MBV was defined as the volume of strong uptake that exceeded the threshold SUV. It represented a high inflammatory activity spot in ARONJ and was measured by the GI-BONE software.

### Pilot study

Prior to comparing SUVmax or MBV of ARONJ among patients, we measured the SUVmax of the bilateral cranial bone to assess the discrepancy between each patients’ normal bone and the reproducibility of SUVmax as calculated by the SPECT quantitative analysis software, by setting the VOI within a 12-pixel (3.96 cm) cube. Simultaneously, we investigated the SUVmax of bilateral cranial bones to assess whether it could be affected by odontogenic infection. We also recorded the SUVmax of the pre-and post-treatment cranial bone to assess whether it could be affected by anti-inflammatory therapy.

### Adjusted SUVmax and threshold for the MBV

Consequently, we tried to standardise the uptake level of the lesion while considering individual differences in the uptake value of the normal bone. We used mean SUVmax of bilateral cranial bones as the control [C] and tried to adjust the SUVmax of the lesion [L] as depicted in the following equation:

$$ {\text{Adjusted}}\,{\text{SUVmax}}\,\left( {{\text{aSUVmax}}} \right) = \left[ L \right]\;\left( {{\text{SUVmax}}\,{\text{of}}\,{\text{lesion}}} \right) - \left[ C \right]\;({\text{SUVmax}}\,{\text{of}}\,{\text{control}}) $$

The optimum threshold for the volume of interest (VOI) was determined to be mean SUVmax of the right and left cranial bones added by 3 (+ 3).

After GI-BONE calculated the SUVmax of each patient's lesion, we calculated each patient's aSUVmax, threshold SUV for VOI using the above equation, and the MBV for each patient during the 1st and 2nd time of SPECT, respectively.

### Statistical analysis

The correlation between SUVmax of the cranial bone at the 1st and 2nd intra-patient SPECT imaging appointment for each patient was determined by calculating the Spearman’s rank correlation coefficient. The intraclass correlation coefficient (ICC) and coefficient of determination (R^2^) were used to assess the reproducibility of the cranial bone’s SUVmax using GI-BONE. The aSUVmax and MBV of the lesion of the jaw at the 1st and 2nd SPECT imaging appointment were compared using the Wilcoxon signed-rank test. A *p*-value of < 0.05 was considered statistically significant. These data were analysed using the SPSS^®^ 25 (Statistical Package for the Social Sciences, IBM Corp., NY, U.S.A) software.

### Ethical approval

The protocol of this study was approved by the Institutional Review Board of Hokkaido University Hospital Ethics Review Board (clinical study number 012-0111). This study was performed in accordance with the 1964 Declaration of Helsinki. All the patients signed a written informed consent.
